# Magnitude Assessment of Adult Neurogenesis in the *Octopus vulgaris* Brain Using a Flow Cytometry-Based Technique

**DOI:** 10.3389/fphys.2018.01050

**Published:** 2018-08-02

**Authors:** Anna Di Cosmo, Carla Bertapelle, Antonio Porcellini, Gianluca Polese

**Affiliations:** Department of Biology, University of Naples Federico II, Naples, Italy

**Keywords:** *Octopus vulgaris*, adult neurogenesis, lophotrochozoan brain, BrdU, flow cytometry

## Abstract

Adult neurogenesis is widespread among metazoans, it occurs in animals with a network nervous system, as cnidarians, and in animals with a complex and centralized brain, such as mammals, non-mammalian vertebrates, ecdysozoans, and a lophotrochozoan, *Octopus vulgaris*. Nevertheless, there are important differences among taxa, especially in the number of the regions involved and in cell proliferation rate during the life-cycle. The comparative evaluation of adult neurogenesis among different brain regions is an arduous task to achieve with only stereological techniques. However, in *Octopus vulgaris* we recently confirmed the presence of active proliferation in the learning-memory centers, multisensory integration centers, and the motor centers of the adult brain. Here, using a flow cytometry technique, we provide a method to quantify the active proliferation in octopus nervous system using a BrdU *in vitro* administration without exposing the animals to stress or painful injections usually used. This method is in line with the current animal welfare regulations regarding cephalopods, and the flow cytometry-based technique enabled us to measure adult neurogenesis more quickly and reliably than histological techniques, with the additional advantage of processing multiple samples in parallel. Flow cytometry is thus an appropriate technique for measuring and comparing adult neurogenesis in animals that are in a different physiological and/or environmental contexts. A BrdU immunoreactivity distribution, to define the neurogenic areas, and the effective penetration *in vitro* of the BrdU is also provided.

## Introduction

Adult neurogenesis is a process consisting of proliferation, migration, and differentiation of newborn cells, which then become functionally integrated into the existing neural circuitry of the adult brain (Duan et al., 2008; Sun et al., 2011). It takes place in defined neurogenic zones which are brain areas exhibiting a high degree of structural plasticity where proliferating neural progenitors produce new cells throughout the entire life of the organisms (Grandel and Brand, 2013).

The adult neurogenic process is widespread among metazoans, given that it occurs both in animals with a network nervous system, for example cnidarians (Galliot et al., 2009), and in animals with complex centralized brains: these include humans and other mammals (Ninkovic et al., 2007; Bergmann et al., 2015), non-mammalian vertebrates (Kaslin et al., 2008), ecdysozoans (Cayre et al., 2002), and lophotrochozoans (Polese et al., 2016; Bertapelle et al., 2017). However, there are important differences in neurogenesis among taxa, particularly with respect to the number of regions involved (Cayre et al., 2002; Lindsey and Tropepe, 2006; Grandel and Brand, 2013) and the cell proliferation rate during the life-cycle also (Lindsey and Tropepe, 2006; Amrein et al., 2011).

Adult neurogenesis goes on throughout life, although the sophisticated balance of multiple factors, such as growth factors, hormones, neurotransmitters, is altered by senescence (Klempin and Kempermann, 2007; Couillard-Després et al., 2011). The latter results in a reduced number of stem cells and cell precursors (Hamilton et al., 2013), in a decrease of their proliferation rate and in a compromised development of new neurons, which taken together lead to a substantial decline in neurogenesis (Riddle and Lichtenwalner, 2007; Couillard-Després et al., 2011; Capilla-Gonzalez et al., 2015).

Considering the variation in lifespan and in time scales of aging, it seems difficult to normalize adult neurogenesis across taxa (Lindsey and Tropepe, 2006). Among mammals, short-lived species are characterized by rapid senescence and a high cell proliferation rate (Amrein et al., 2011). In contrast, long-lived species demonstrate more gradual senescence and a slower proliferation rate (Amrein et al., 2011; Hamilton et al., 2013). The comparison of absolute age shows that both short- and long-lived species are affected by an exponential decline in proliferation, occurring mostly between young and middle age (Barker et al., 2011). The areas implicated in adult neurogenesis in the mammalian brain (Fuchs and Flügge, 2014) are the sub-ventricular zone, from where neuroblasts migrate to the olfactory bulbs, which are involved in olfactory memory formation, odorant discrimination and social interactions (Ming and Song, 2011), and the sub-granular zone of the hippocampal dentate gyri, which are implicated in learning and spatial memory (Ernst and Frisén, 2015; Yau et al., 2015).

A different scenario occurs in non-mammalian vertebrates such as birds, reptiles, amphibians, and teleosts, in whose brains adult neurogenesis is more diffuse (Grandel and Brand, 2013) and widespread, but is more pronounced in comparison to mammals (Lindsey and Tropepe, 2006).

In the avian brain proliferating cells are located in the ventricular zone of the forebrain (Alvarez-Buylla et al., 1990, 1992, 1998) and migrate to specific telencephalic sites (Grandel and Brand, 2013).

In reptiles, adult neurogenesis contributes to the brain enlargement observed with the age (Marchioro et al., 2005) and takes place in several areas of the telencephalon. The nervous systems of amphibians and teleosts are characterized by more neurogenic compartments than is described in other vertebrates (Raucci et al., 2006; Zupanc and Sîrbulescu, 2011; D’Amico et al., 2013; Ganz and Brand, 2016). In zebrafish, neurogenic compartments are distributed along the entire rostro-caudal axis of the brain which ensures the availability of new neurons throughout life to replace cells lost after injury (Kizil et al., 2012).

Studies on adult neurogenesis in invertebrates are few in comparison to those in vertebrates (Lindsey and Tropepe, 2006). The process has been investigated only in few taxa, such as cnidarians (Galliot et al., 2009; Galliot and Quiquand, 2011), ecdysozoans (Cayre et al., 2002; Dufour and Gadenne, 2006; Schmidt and Derby, 2011; Fernández-Hernández et al., 2013; Benton et al., 2014), and recently in the lophotrochozoan mollusc: *Octopus vulgaris* among cephalopods (Bertapelle et al., 2017), and *Cipangopaludina chinensis* among gastropods (Swart et al., 2017). In cnidarians, which lack a centralized brain, proliferation, migration and differentiation occur: the interstitial stem cells of body column proliferate, providing progenitors for neurons that migrate to the dense nerve nets located in apical and basal regions, as described in *Hydra* polyps (Galliot and Quiquand, 2011).

In ecdysozoan taxa, the process is restricted to specific compartments of the brain: mushroom bodies of insects and the lateral-medial soma clusters of the crustacean olfactory pathway (Schmidt and Harzsch, 1999; Cayre et al., 2000, 2007; Schmidt and Derby, 2011). In the lophotrochozoan *O. vulgaris*, adult neurogenesis is mainly located in specific lobes of the SUP including the vertical frontal system, optic tract lobes and the OL (Bertapelle et al., 2017). Interestingly, the neurogenic process in adult *O. vulgaris* is affected by environmental stimuli (Bertapelle et al., 2017).

The model of the neurogenesis emerging from adult insect studies describes a persistent cluster of proliferating cells in the mushroom bodies (Cayre et al., 2007). Newborn interneurons push old cells to the outer layer of cortex, increasing cell density (Cayre et al., 1997; Scotto-Lomassese et al., 2002), implying a constant reorganization of neural circuits throughout life (Cayre et al., 2002, Cayre et al., 2006, 2007; Malaterre et al., 2002). A completely different model is found in the crustacean brain, where proliferation occurs in cell niches located in two different clusters of the integrative sensory areas (Sandeman and Sandeman, 2000). Active proliferation again suggests that the continual turnover of olfactory interneurons may be linked to the turnover of olfactory circuits (Sullivan and Beltz, 2005). The niche cell population appears not to be self-renewing and some histological evidence suggests that cell precursors have a hematopoietic origin, due to intimate connections of the niches with the blood vessels (Benton et al., 2014; Hartenstein, 2014; Chaves da Silva et al., 2015). To date, the few data about neurogenic events in lophotrochozoans refer to regeneration after injury as described in planarians (Cowles et al., 2013), annelids (Meyer and Seaver, 2009), and gastropods (Matsuo et al., 2012).

The occurrence of adult neurogenesis in cephalopods was demonstrated in the brain of *O. vulgaris* in which cell proliferation and synaptogenesis following intellectual, sensory and motor stimulation (Bertapelle et al., 2017). The *O. vulgaris* brain is located around the esophagus, in a cartilaginous “cranium” between the eyes, and it consists in a supra-oesophageal and sub-oesophageal masses connected to two OL, and it is characterized by a hierarchical organization (Young, 1971, 1977; Wells, 1978; De Lisa et al., 2012; Shomrat et al., 2015). *O. vulgaris* has a short life cycle, unlike other molluscs, most cephalopods “live fast and die young” (Powell and Cummins, 1985; O’Dor and Wells, 1978). *O. vulgaris* females live 1 year or rarely 2, during which time they grow very fast and reproduce. After mating, the female spawns and spends all its energies in maternal care: it refrains from feeding and spends its whole time in cleaning and ventilating the eggs, eventually dying of starvation. The male mates several times during its life and may live longer than the female (Di Cosmo and Polese, 2014; Polese et al., 2015).

Here, for the first time in Lophotrochozoa, we quantify adult neurogenesis in specific areas of the *O. vulgaris* brain, using a flow cytometry techniques based on BrdU incorporation (Taupin, 2007). BrdU is a synthetic nucleoside, analog of thymine, incorporated into newly synthesized DNA during the S-phase of the cell cycle (Nowakowski et al., 1989), and largely used in proliferation assays (Kee et al., 2002) because it is an excellent specific marker of neurogenesis. Using the same specific marker, we performed a quantitative fluorescence-based cytometry assay on dissociated cells from brain areas previously identified as adult neurogenic sites by BrdU immunoreactivity distribution. To perform the flow-cytometry assay we developed a novel and appropriate protocol to dissociate octopus brain specific neurogenic areas (Maselli et al., 2018). The choice of an *in vitro* BrdU administration is to avoid any kind of stressful manipulation that could interfere with the animal physiological status affecting somehow neurogenic processes (Bertapelle et al., 2017).

## Materials and Methods

### Animals

Specimens of *O. vulgaris* [*n* = 7 (three male and four female), weight 800–1000 g], collected from the wild in the Bay of Naples, were maintained in aquarium tanks for 3 days (Polese et al., 2014; Di Cosmo et al., 2015). All specimens were sexually mature and before spawning. Our research conformed to the European Directive 2010/63 EU L276, the Italian DL. 4/03/2014, n. 26 and the ethical principles of Reduction, Refinement and Replacement (protocol n. 0124283-08/11/2012 approved by the University Ethical Committee and the Italian Ministry of Health). Octopuses were euthanized by isoflurane overdose (Polese et al., 2014) and brains were dissected in sterile conditions. No attempt was made to induce neurogenesis in these specimens.

### BrdU Immunohistochemistry

Dissected brains (*n* = 2: one male and one female) masses: central brain SUP, SUB, and OL were exposed *in vitro* (Cayre et al., 1996) to BrdU (Sigma–Aldrich, St. Louis, MO, United States) (30 μg/ml) to a final concentration of 0.1 mM in cell culture medium (Maselli et al., 2018) for 1 h in an incubator at saturation humidity at 18°C, then fixed in Bouin’s fluid for 24 h at room temperature, dehydrated in ethanol, cleared in Bioclear and embedded in paraffin. Sections (7 μm) were cut on a microtome and mounted on albumin-coated slides, then cleared, rehydrated and incubated with HCl 1N for 30 min to allow DNA denaturation. After several rinses (4 for 10′ each in PBS), sections were incubated for 20 min with 1% normal horse serum (Life Technologies, Carlsbad, CA, United States) and then incubated in anti-BrdU (dilution 1:1000, clone BU-33, Cat# B8434, **RRID**:AB_476811, from Sigma–Aldrich, St. Louis, MO, United States) at 4°C overnight in a humid chamber. After many washes in PBS (4 of 10′ each), sections were incubated with horse anti-mouse secondary antibody biotin conjugated (dilution 1:200, from ThermoFisher Scientific, Waltham, MA, United States), for 1 h at room temperature, then rinsed in PBS (2 of 10′ each) and incubated with streptavidin conjugated to horseradish peroxidase (dilution 1:200, from Life Technologies Carlsbad, CA, United States) for 1 h at room temperature. 3% DAB (3.30-diaminobenzidine tetrahydrochloride, Sigma–Aldrich, St. Louis, MO, United States) with 0.03% hydrogen peroxide in Tris buffer (0.05 M, pH 7.6) was used as chromogen and sections were dehydrated and mounted in Permount (ThermoFisher Scientific, Waltham, MA, United States). In controls, sections from a brain of octopus that had not received BrdU incorporation (*n* = 1 female) were treated for labeling with anti-BrdU as well. Using imageJ software (version 1.48, National Institute of Health, New York, NY, United States), the background signal detected in negative controls was subtracted to evaluate the BrdU positive cells. The BrdU-ir was detected using a Leica DM-RB microscope.

### Preparation of Samples for Flow Cytometry

Dissected brains (*n* = 3: two males and one female) masses (SUP, SUB, and OL) were exposed *in vitro* (Cayre et al., 1996) to BrdU (Sigma–Aldrich, St. Louis, MO, United States) in cell culture medium (Maselli et al., 2018) (30 μg/ml) to a final concentration of 0.1 mM for 1 h in an incubator at saturation humidity at 18°C. Cells from a brain of octopus (*n* = 1 female) that had not received BrdU incorporation were used as negative control.

### Cell Dissociation

**FIGURE 1:**
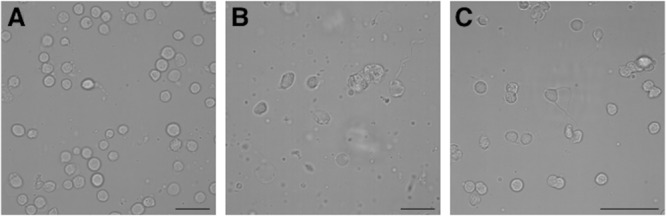
Photomicrographs of *Octopus vulgaris* healthy neurons freshly dissociated from different brain areas before to perform flow cytometry assay: **A** – optic lobe neurons; **B** – suboesophageal mass neurons; **C** – supraesophageal mass neurons (scale bar = 50 μm).

Suboesophageal mass, SUB, and OL were separately minced with a scalpel, incubated with 1 mg/ml papain enzyme (Sigma–Aldrich, St. Louis, MO, United States) in artificial sea water (ASW) for 30 min at room temperature and then incubated with 1 mg/ml trypsin (Sigma–Aldrich, St. Louis, MO, United States) in ASW for 20 min at room temperature. Samples were extensively washed in Leibovitz-15 medium (ThermoFisher Scientific, Waltham, MA, United States) to stop the enzyme function and triturated with 1 ml and 0.200 ml pipette tips to yield single cells until no cell cluster were visible (Maselli et al., 2018). Dissociated cells were checked with an inverted microscope (**Figure 1**), counted in a Burker chamber and centrifuged at 6 × *g*, fixed in cool 70% ethanol and stored at -20°C.

### Flow Cytometry

Cells were extensively washed and incubated with HCl 2N for 30 min, rinsed twice (10′) in phosphate/citrate buffer at pH 7.4 and incubate with anti-BrdU-FITC (10 μl of anti-BrdU-FITC per 10^6^ cells) (clone B44, Cat# 347583, **RRID**: AB_400327, from Becton, Dickinson and Company BD Biosciences, San Jose, CA, United States) in ASW and BSA 1% for 30 min, raised against a iodouridine-conjugated ovalbumin, that recognizes 5-bromo-2′deoxyuridin and iodouridine in single-stranded DNA. After washes, the cells were stained with PI (10 μg/ml) in ASW Triton 0.1% with RNAse A (100 μg/ml). Flow cytometry was performed using a BD Accuri C6 flow cytometer (Becton, Dickinson and Company BD Biosciences, San Jose, CA, United States). Doublets and cell aggregates were subtracted on the basis of pulse shape (pulse peak vs. pulse area analysis) and by applying the aggregation model in the cell cycle analysis (Wersto et al., 2001). Bivariate analysis of BrdU content FITC versus DNA content PI were performed using FlowJo software (FlowJo LLC, Ashland, OR, United States). Experiments were performed in triplicate. The background signal was based on data collected from a brain that was not incorporated with BrdU and gates were set manually by using control samples. All data are reported as percentage ± standard deviation (s.d.).

## Results

### Distribution of BrdU Immunoreactivity

**FIGURE 2:**
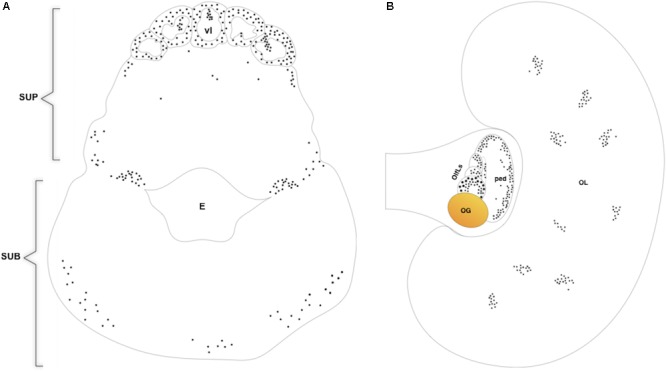
Diagram of BrdU immunoreactivity distribution on transversal section of *O. vulgaris* supra and suboesophageal masses **(A)**, horizontal section of *O. vulgaris* optic lobe and optic lobe tract **(B)**. SUP, supraoesophageal mass; vl, vertical lobe; E, esophagus; SUB, suboesophageal mass; OL, optic lobe; OlfLs, olfactory lobules; OG, optic gland; ped, peduncle lobe.

**FIGURE 3:**
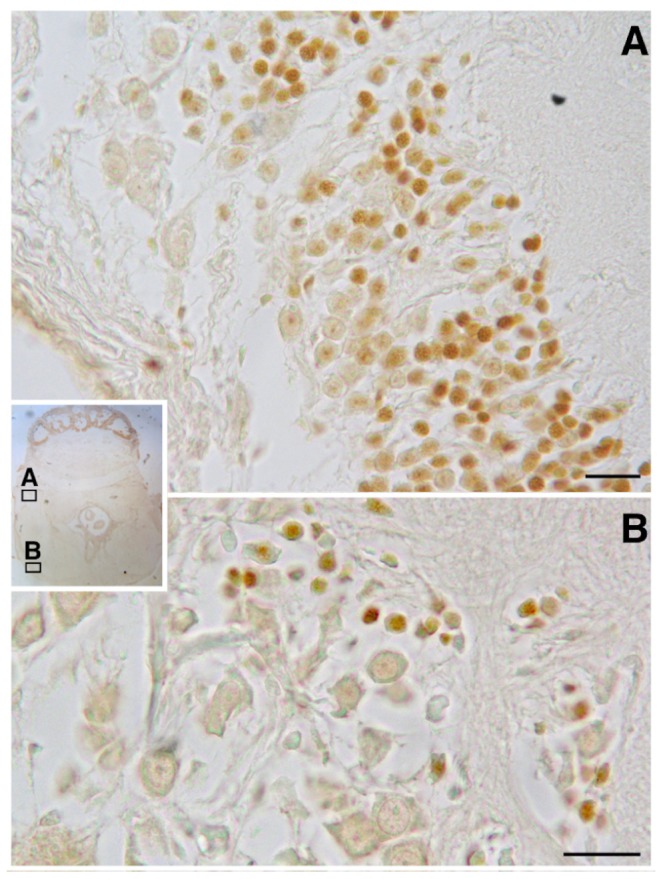
BrdU immunoreactivity on transversal section of *O. vulgaris* central nervous system suboesophageal mass: **A** – palliovisceral lobe showing several interneuron nuclei labeled; **B** – posterior pedal lobe with few scattered immunopositive interneuron nuclei (scale bar = 50 μm).

BrdU immunoreactivity (-ir) was positively located in the neuron nuclei of the specific lobes of the supra-esophageal mass including the optic tract lobes and the OL, and in the sub-esophageal mass (summarized in **Figures 2, 3**). This results non-only confirm and straighten what was previously found using just PCNA immunocytochemistry (Bertapelle et al., 2017), but more over let us to discriminate between neurons and glial cells since that the latter nuclei are exclusively located in the neuropils of all lobes of the CNS (Young, 1971) and never appear labeled with anti BrdU. An example of BrdU-ir staining is shown in **Figure 3**. The BrdU immunoreactivity in both specimens (one male and one female) showed an overlapping distribution that is summarized in the **Table 1**. In control sections, no specific labeling was observed, as also on randomly picked sections from BrdU treated brains where the primary antibody against BrdU was substituted with plain PBS to control secondary unspecific staining.

**Table 1:** BrDU immunoreactivity distribution.

Brain regions		Functions
***Subesophageal mass***		
Brachial	–	Intermediate and lower motor centers: motor
Anterior pedal	–	coordination – control of movement and
Lateral pedal	+	visceral functions
Posterior pedal	+	
Palliovisceral	+	
***Supraesophageal mass***		
Anterior basal	–	Higher motor centers:
Median basal	–	motor coordination – motor control
Dorsal basal	+	
Posterior buccal	+	Tactile discrimination – Predatory and
Superior buccal	–	exploratory behaviors –
Median inferior frontal	+	Nociceptive information
Subfrontal	+	
Lateral superior frontal	+	Cognitive centers: Learning – Memory storage
Median superior frontal	+	and consolidation – Elaboration of motor
Subvertical	+	program
Vertical	+	
***Optic lobe***		
Outer granular layer	–	Memory storage – Integration and coordination
Inner granular layer	–	of sensory stimuli – Genesis of motor action
Medulla	+	pattern
***Optic tract lobes***		
Olfactory lobes	+	Integration of olfactory stimuli – Endocrine
Peduncle	+	control of reproduction
Optic gland	–	

*Presence (+)/Absence (-) of neurogenesis.*

### Flow-Cytometry/Bivariate Analysis

**FIGURE 4:**
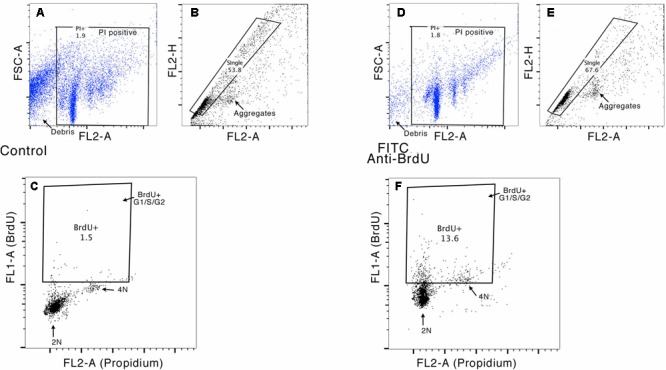
Example of the gating strategy for flow cytometry analysis. In this sample gating performed on OL neurons, cells were first gated for Propidium Iodide staining, to exclude debris (FL2-A vs. FSC-A, see panels **A** and **D**) and then gated for singlets (FL2-H vs. FL2-A, see panels **B** and **E**), to exclude cell doublets. This cell population was further analyzed for their uptake of BrdU versus DNA content (FL1-H vs. FL2A). Panels **C** and **F** show the gates used to evaluate the percentage of cells in S and G2/M phase (stained with PI) and to measure the percentage of BrdU-positive cells (DNA content ranging between G1 and G2).

Bivariate analysis of BrdU content (FITC) versus DNA content (PI) was performed in order to discriminate between cells in G2/M or S-phase that incorporate BrdU during *de novo* DNA synthesis, and cell doublets or cells that incorporate BrdU for DNA repair. Furthermore, given the occurrence of polyploidy in molluscan nervous systems (Matsuo et al., 2012; Yamagishi et al., 2012), doublets and cell aggregates were subtracted on the basis of pulse shape (pulse peak vs. pulse area analysis) (van Oven and Aten, 1990) and the bivariate analysis that allowed us to exclude G0/G1 cells with DNA content higher than 2 n that overlaps the S/G2 peaks and are difficult to distinguish on the histogram of DNA content analysis carried out with PI alone. **Figure 4** shows an example of gating strategy performed on OL neurons.

**FIGURE 5:**
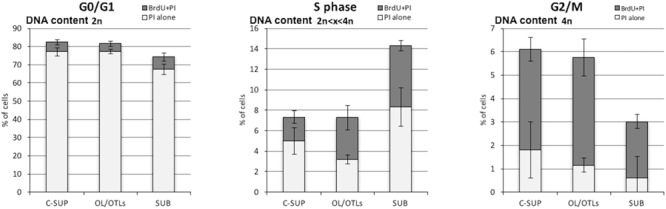
Percentage of PI positive (white) and PI-BrdU double positive (gray) neurons within the three different cell cycle phases in each brain areas considered.

The proportion of BrdU positive cells (*duplicating and self DNA repairing*) in the supraesophageal mass without OL is 11.98 ± 1.2%, showing a DNA content compatible with G1, S, and G2 cell cycle phases. Double positive (BrdU and PI) cells are 6.63 ± 0.75%, their DNA content is higher than 2N, according to S and G2/M phase (**Table 2**). BrdU positive cells in the OL and optic tract lobes are 13.0 ± 0.88%, showing a DNA content compatible with G1, S, and G2 cell cycle phases. Double positive (BrdU and PI) cells are 8.6 ± 1.03%, showing a DNA content higher than 2N, compared with S and G2/M phases (**Table 2**). BrdU positive cells in the SUB make up 15.5 ± 2.55% of the cells, showing a DNA content compatible with G1, S, and G2 cell cycle phases. Double positive (BrdU and PI) cells make up 8.35 ± 0.75%, showing a DNA content higher than 2N, according to S and G2/M phases. In **Figure 5** it is shown the percentage of PI positive and PI-BrdU double positive neurons within the three different cell cycle phases in each brain areas considered.

**Table 2:** Percentage of BrdU+ and PI+ cells among brain areas.

Brain areas		BrdU + cells (%)	PI + cells (DNA content > 2N) (%)
Supraesophageal mass: central (SUP) and lateral (OL/OTLs)	SUP OL/OTLs	11.98 ± 1.2 13.0 ± 0.88	6.63 ± 0.75 8.6 ± 1.03
Subaesophageal mass	SUB	15.5 ± 2.55	8.35 ± 0.75

## Discussion

Besides confirming adult neurogenesis in the lophotrochozoan clade, this study has quantified the extent of active cell proliferation in the brain of adult *O. vulgaris* using a flow cytometry assay. Given its complex nervous system, which is comparable to mammalian and insect brains (Katz, 2016), octopus becomes the most suitable candidate to study adult neurogenesis among lophotrochozoans (Bertapelle et al., 2017). The functional role of adult neurogenesis is not fully understood, however, it is indisputably involved in higher cognitive capabilities (Kempermann, 2002; Cameron and Christie, 2007; Braun and Jessberger, 2014), processing of sensory information (Nissant et al., 2009; Breton-Provencher and Saghatelyan, 2012; Cheetham et al., 2016) and thus to improve adaptation to environmental changes (Glasper et al., 2012; Opendak and Gould, 2015; Liscovitch-Brauer et al., 2017), features extensively exhibited by *O. vulgaris* (Bertapelle et al., 2017). Going beyond our previous data obtained with PCNA (Bertapelle et al., 2017) here, we used a more reliable marker of cell proliferation, the BrdU, in both immunocytochemistry analysis and in flow cytometry assay, the latter for a rapid quantification of adult neurogenesis.

To carry out this research we developed an appropriate cell dissociation protocol for the octopus brain (Maselli et al., 2018) and applied it to specific neurogenic sites. This allowed us to assess the magnitude of the adult neurogenic event in octopus by the flow-cytometry assay based on a bivariate analysis of incorporated BrdU versus DNA content in neurons.

The bivariate analysis using PI and conjugated anti-BrdU FITC allowed us to discriminate proliferating cells from non-proliferating cells (Kim and Sederstrom, 2015).

Furthermore, aneuploid and tetraploid neurons in G0/G1 with DNA content >2N were excluded from cell cycle analysis since they were unable to incorporate BrdU in a fast pulse administration (**Figure 5**).

The advantage of this assay results in a faster and more accurate quantitative analysis if compared with the counting of BrdU-ir cells performed at microscope level (Bilsland et al., 2006; Spoelgen et al., 2011). The BrdU immunocytochemistry analysis though, still retain its essential function in localizing the proliferating cells labeling the neurogenic areas. Furthermore, in octopus, where it is well known the anatomical distribution of neurons and glial cells, the BrdU immunocytochemistry is fundamental to discriminate between them given that the latter are exclusively located in the neuropils of all lobes of the CNS (Young, 1971). At last, the combination of both immunocytochemistry analysis and flow cytometry assay resulted absolutely necessary to give an accurate characterization of adult neurogenesis in *O. vulgaris.*

The effective proliferating neurons detected in different *O. vulgaris* brain areas revile that neurogenic events appear in a comparable measure in both cognitive centers and motor centers (**Table 2**) (Bertapelle et al., 2017).

Tracing a parallel between *O. vulgaris* and mammals we observed that the effective proliferating cells detected in octopus supraesophageal mass are mainly located in the vertical frontal system, basal and buccal lobes considered analogous to mammalian hippocampus (Young, 1991) in which adult neurogenesis occurs (Zhang et al., 2015).

In SUB, the lobes that appear to be BrdU-ir (**Table 1**) with a discrete percentage of effective proliferating cells (**Table 2**) are the pedal and palliovisceral lobes that are the intermediate motor centers in charge of controlling and modulating the lower motor centers that supply the muscle directly (Wells, 1978; Boycott, 1961). In spite of this the occurrence of adult neurogenesis in the SUB, that are considered as equivalent to the vertebrate spinal cord (Boycott and Young, 1950), could be related to a potential mechanism of response to a novel stimulus and for habituation to repeated mechanosensory exposures (Shechter et al., 2011). Furthermore, in mammals, comparative analysis conducted on different species revealed that short-lived species had more extensive hippocampal proliferation than long-lived species (Amrein and Lipp, 2009; Amrein et al., 2011). The fast life cycle of *O. vulgaris* (Hanlon and Messenger, 1998; Anderson et al., 2002) may be comparable to short lived mammalian species allowing us to interpret the adult neurogenic events of octopus brain as required features necessary to enhance the plasticity needed to face changing environmental challenges.

Thus, avoiding any undesired effect due to the classical stressful BrdU injection, our experimental approach results appropriate for further study on *O. vulgaris* aimed to evaluate the effects of varied physiological and/or environmental contexts on adult neurogenesis.

## Author Contributions

ADC and CB conceived the experiments. CB and GP performed the experiments and acquired the data. AP acquired and analyzed the flow-cytometry data. ADC and GP analyzed and interpreted the data. ADC obtained funding. All authors participated in writing, reading and approving the manuscript.

## Conflict of Interest Statement

The authors declare that the research was conducted in the absence of any commercial or financial relationships that could be construed as a potential conflict of interest.

## Abbreviations

ASW: artificial seawater
BrDU: bromodeoxyuridine
BSA: bovine serum albumin
FITC: fluorescein isothiocyanate
G0: gap 0 phase of the cell cycle
G1: gap 1 phase of the cell cycle
G2: gap 2 phase of the cell cycle
-ir: immunoreactivity
KLH: keyhole limpet hemocyanin
M: mitotic phase
OL: optic lobes
PBS: phosphate buffered saline
PCNA: proliferating cell nuclear antigen
PI: propidium iodide
RPM: revolutions per minute
S: synthesis phase
SUB: suboesophageal mass
SUP: supraesophageal mass
