# Corrigendum: Magnitude Assessment of Adult Neurogenesis in the *Octopus vulgaris* Brain Using a Flow Cytometry-Based Technique

**DOI:** 10.3389/fphys.2019.00076

**Published:** 2019-02-07

**Authors:** Anna Di Cosmo, Carla Bertapelle, Antonio Porcellini, Gianluca Polese

**Affiliations:** Department of Biology, University of Naples Federico II, Naples, Italy

**Keywords:** *Octopus vulgaris*, adult neurogenesis, lophotrochozoan brain, BrdU, flow cytometry

In the original article, there was a mistake in Figure 3 as published. The incorrect image was used. The corrected Figure 3 appears below.

**Figure 3:**
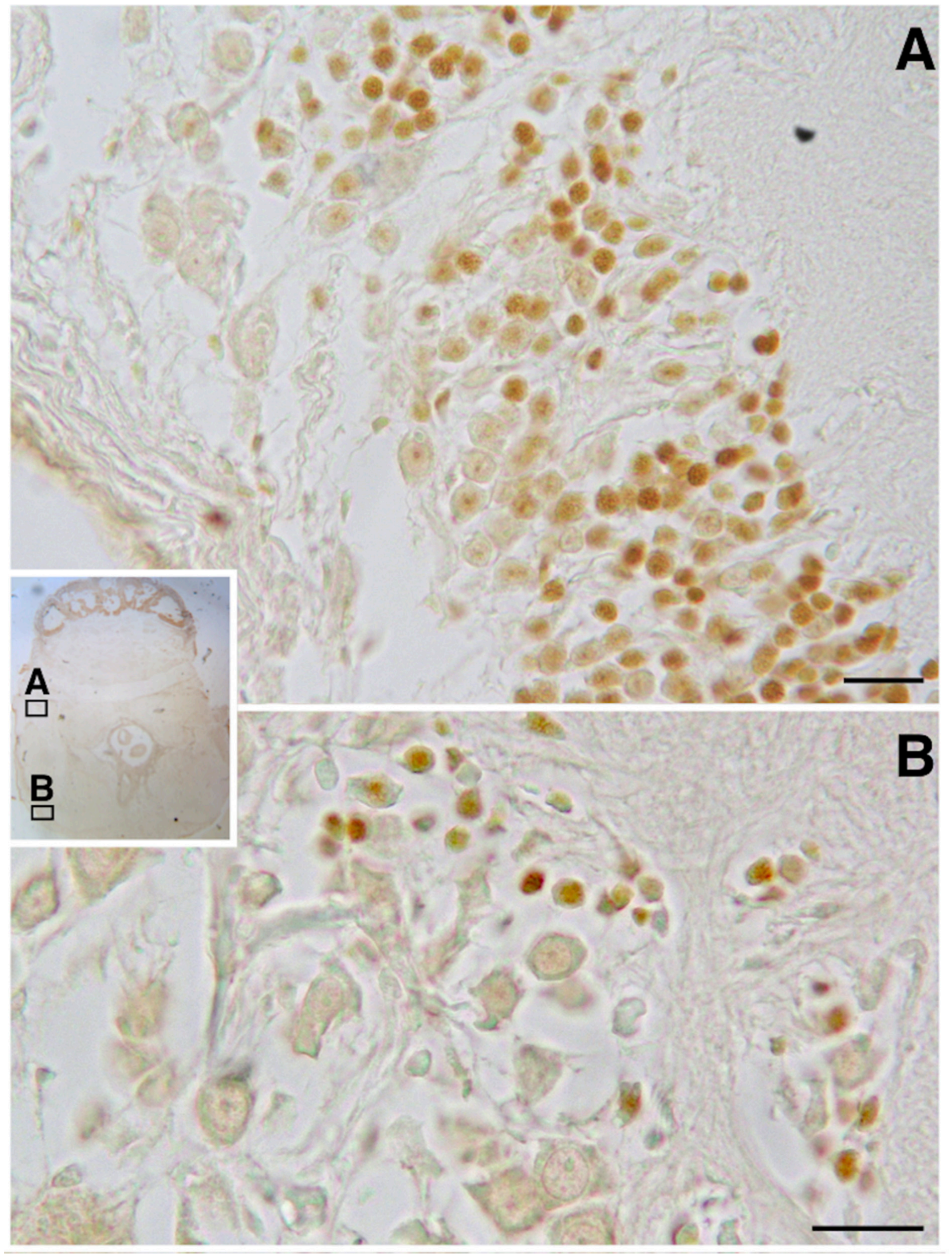
BrdU immunoreactivity on transversal section of *O. vulgaris* central nervous system suboesophageal mass: **A** – palliovisceral lobe showing several interneuron nuclei labeled; **B** – posterior pedal lobe with few scattered immunopositive interneuron nuclei (scale bar = 50 μm).

The authors apologize for this error and state that this does not change the scientific conclusions of the article in any way. The original article has been updated.

## Conflict of Interest Statement

The authors declare that the research was conducted in the absence of any commercial or financial relationships that could be construed as a potential conflict of interest.

